# Necroptosis-Related miRNA Biomarkers for Predicting Overall Survival Outcomes for Endometrial Cancer

**DOI:** 10.3389/fgene.2022.828456

**Published:** 2022-05-27

**Authors:** Hualin Song, Tianjie Li, Jindong Sheng, Dan Li, Xiangyu Liu, Huiting Xiao, Hu Yu, Wenxin Liu, Ke Wang, Ying Chen

**Affiliations:** ^1^ Department of Gynecological Oncology, Tianjin Medical University Cancer Institute and Hospital, National Clinical Research Center for Cancer, Tianjin, China; ^2^ Key Laboratory of Cancer Prevention and Therapy, Tianjin, China; ^3^ Tianjin’s Clinical Research Center for Cancer, Tianjin, China; ^4^ Department of Obstetrics and Gynecology, Beijing Friendship Hospital, Capital Medical University, Beijing, China

**Keywords:** necroptosis, miRNA, endometrial cancer, prognosis, biomarker

## Abstract

Endometrial cancer (EC) is the gynecological tumor with the highest incidence. In recent years, it has been proved that necroptosis is a method of cell death related to EC. However, the expression of necroptosis-related miRNA in EC and its correlation with prognosis still ill-defined. Use the Cancer Genome Atlas (TCGA) cohort to obtain prognostic data and related clinical data for ECs and normal endometrium tissues. In this study, we identified three necroptotic regulatory miRNAs that are necroptosis-related and survival-related miRNAs (DENSMs) between normal endometrium tissues and EC from 13 necroptosis-related miRNAs. The three DENSMs signature was built to develop prognostic model and classified all EC patients into a high or low risk group. EC patients in the low-risk group showed significantly higher survival possibilities than those in the high-risk group (*p* = 0.0242), and the risk score was found to be an independent prognosis factor for predicting the OS of EC patients (*p* = 0.0254) in multivariate Cox regression. Gene Ontology (GO) and Kyoto Encyclopedia of Genes and Genomes (KEGG) analysis showed dephosphorylation, microtubule, protein serine/threonine kinase activity, PI3K-Akt signaling pathway and MAPK signaling pathway are closely related to it. In conclusion, the risk prediction model based on necroptosis-related miRNAs can effectively predict the prognosis of EC patients.

## Introduction

Endometrial cancer (EC) is the most common gynecological cancer in developed countries and ranks second in developing countries (Bray et al., 2018). In 2018, there will be an estimated 382,000 new cases and 89,900 deaths worldwide (Bray et al., 2018). With the decline in birth rate and the prevalence of obesity, the incidence rate has risen significantly (Bray et al., 2018). Most of the primary cases of EC patients were surgically removed, and the prognosis was good, with a 5-years survival rate of more than 70% (Uccella et al., 2016). However, vaginal or pelvic EC recurrence, usually metastasized to a distant location, still occurs 15%–20% of cases after surgery (Connor and Rose, 2018). Despite progress in surgical techniques, chemotherapy or radiation, the data show an increasing mortality rate of EC (Balch et al., 2010; Siegel et al., 2021; Sung et al., 2021). Therefore, a thorough prognostic assessment is urgently needed. Recent studies have shown that necroptosis plays an important role in the formation and development of EC, suggesting that necroptosis-related miRNAs can be used as prognostic markers of EC (Ghanekar and Sadasivam, 2019; López-Janeiro et al., 2021).

Necroptosis is a cell death caused by regulation of independent of caspase. Necroptosis is mainly composed of protein kinase 1 receptor (RIPK1), protein kinase 3 receptor (RIPK3) and mixed lineage kinase domain-like protein (MLKL) (Choi et al., 2019) (Choi et al., 2019). Necroptosis is a programmed necrosis (Galluzzi and Kromer, 2008; Christofferson and Yuan, 2010; Wallach et al., 2016; Galluzzi et al., 2018; Green 2019). The morphological characteristics of necrotic apoptotic cells include crisis of intracellular energy metabolism, incomplete cell membrane and release of inflammatory factors (Pasparakis and Vandenabeele, 2015). Necroptosis plays an important role in the emergence and development of the metastasis of cancer, cerebrovascular diseases and a variety of diseases (Galluzzi et al., 2017; Choi et al., 2019; Tang et al., 2020; Xia et al., 2020). Necroptosis has positive and negative effects in malignant tumors (Su et al., 2015; Galluzzi et al., 2018; Frank and Vince, 2019; Liu et al., 2021). On the one hand, necroptosis can promote tumor metastasis and progression alone or in combination with other factors, and on the other hand, necroptosis acts as a protection against tumor development when apoptosis is disrupted (Su et al., 2015; Frank and Vince 2019; Liu et al., 2021). In this study we examined the influence of necroptosis on the prognosis of ECs.

MicroRNAs (miRNAs) are short non-coding RNAs 19-24 nucleotides in length that play a role in regulating gene expression through antisense complementation or specific mRNA complementation (Vasilatou et al., 2015). Many studies have shown that a large number of miRNAs are abnormally expressed in a large number of tumors and participate in the occurrence and development of tumors as oncogenes or tumor suppressor genes (Bueno et al., 2008; Torres et al., 2013). Previous research tried to establish a connection between miRNA and EC. For example, has-mir-337-3p, let-7b, and miR135a are considered to have the potential as non-invasive biomarkers (Sun et al., 2017). Although there have been many reports on the influence of miRNAs on the formation and development of EC (Sun et al., 2017; Jiang et al., 2018; Sun et al., 2018; Wang Q. et al., 2020), there are no reports on the use of necroptosis miRNAs as molecular markers to predict the prognosis of EC patients. Therefore, whether the miRNAs associated with necroptosis is related to the prognosis of patients with EC remains unclear. It is very important to study miRNAs associated with necroptosis as molecular markers to predict the prognosis of EC. However, there are few studies on the prognostic value of miRNAs associated with necroptosis in EC and even other cancers (Chen et al., 2022; Zhang et al., 2022). Zhang et al. reported that necroptosis-related signatures as an independent prognostic model for head and neck squamous cell carcinoma (Zhang et al., 2022). Chen et al. showed they used necroptosis-related lncRNA to establish novel prognostic signature and predict the immunotherapy response in breast cancer (Chen et al., 2022).

To solve the above problems, in this study we obtained the clinical information and miRNAs profile of normal endometrium and EC tissue from the Cancer Genome Atlas (TCGA). From previously published literature, we have identified and classified necroptosis-related miRNAs (Liu et al., 2021). In this study, we developed a prognostic model to understand the expression levels of miRNA necroptosis in EC and normal endometrial tissues, and to study the prognostic value of miRNAs. Kaplan-Meier curve (KM), area under the curve (AUC), and forest plot show that these models have good predictive effects. Finally, predict the target gene by the different miRNAs expressed, and analyze the functional enrichment of the target gene to study the potential mechanism of these miRNAs.

## Materials and Methods

### Raw Data Acquisition and Pre-processing

As of 31 August 2021, we have obtained miRNA and clinical feature data for 546 EC and 33 normal tissue patients from the TCGA database (https://portal.gdc.cancer.gov/repository). The clinicopathological characteristics of the patients in the TCGA database are shown in Table 1. The list of 13 necrotizing miRNA comes from earlier literature (Liu et al., 2021) and is showed as follow: miR-495, miR-331-3p, miR-15a, miR-148a-3p, miR-7-5p, miR-141-3p, miR-425-5p, miR-200a-5p, miR-210, miR-223-3p, miR-500a-3p, miR-181-5p, miR-16-5p. Then extract th e miRNA expression matrix of necroptosis, and perform data matching, data filtering, and data correction. At the same time, relevant clinical data are also filtered and matched for subsequent use. All data are standardized using the cross-array scaling function of the limma R/Bioconductor package. In addition, our research strictly complies with the TCGA data access policy and publication guidelines. The RNA-seq data of the miRNA target genes were downloaded from the GEO database (https://www.ncbi.nlm.nih.gov/geo/, ID: GSE17025).

**TABLE 1:** clinicopathological features of the EC patients used in this study.

	TCGA cohort	Training cohort	Validation cohort
No. of patients	546	364	182
Age (median, range)	64 (31-90)	63 (31-90)	64 (33-87)
Grade (%)			
G1	99 (18.1%)	61 (16.8%)	38 (20.9%)
G2	122 (22.3%)	77 (21.2%)	45 (24.7%)
G3	313 (57.3%)	218 (59.9%)	95 (52.2%)
unknown	12 (2.2%)	8 (2.2%)	4 (2.2%)
Stage (%)			
I	339 (62.1%)	225 (61.8%)	114 (62.6%)
II	52 (9.5%)	32 (8.8%)	20 (11.0%)
III	124 (22.7%)	83 (22.8%)	41 (22.5%)
IV	30 (5.5%)	23 (6.3%)	7 (3.8%)
unknown	1 (0.2%)	1 (0.3%)	0 (0.0%)
Survival status			
OS days (median, range)	830 (0-6,859)	854 (0-5,651)	762 (6-5,859)
censored (%)	465 (85.2%)	309 (84.9%)	156 (85.7%)

### Development and Validation of the Necroptosis-Related miRNAs Prognostic Model

To accurately assess the prognostic value of necroptosis-related miRNAs, we used the “Limma” package to find differentially expressed necroptosis-related miRNAs. The different expressed miRNAs were filtered according to the standards (|log2FC| ≥ 0 and FDR <0.05). Then, KM curves were used to draw survival curves for the differentially expressed necroptosis-related miRNAs, in order to find the differentially expressed necroptosis-related and survival-related miRNAs (DENSMs). We finally identified a total of three DENSMs for the development of prognostic models and the next step of analysis. Then we divided the patient data of TCGA into two groups according to the ratio of 2:1, one is training cohort for building the prognostic model, and the other is validation cohort for model validation. A total of 364 patients were assigned to the training cohort and 182 patients were assigned to the validation cohort. The clinicopathological characteristics of the training cohort and validation cohort patients are shown in Table 1. The use of DENSMs to calculate risk scores in training cohort is the key to constructing prognostic models. We extracted many clinical characteristics of EC patients from the TCGA database, including age, grade, and stage. These clinical variable data are combined with risk scores in our prognostic model for further analysis. Then, univariate and multivariate Cox regression models are used to further analyze the risk score to determine whether the risk score can be an independent prognostic factor. The predictive ability of the model is determined by drawing the Receiver Operating Characteristics (ROC) curve to calculate the area under the ROC curve (AUC). The “survminer”, “survival”and “time- ROC” R packages were employed to perform a 1-year, 3-years and 5-years ROC curve analysis. According to the calculation of risk score, we divided training cohort EC patients into low-risk and high-risk subgroups by the median of risk score. KM curve analysis compares the overall survival (OS) time of the two subgroups, and Cox regression compares the risk ratio of the two subgroups. Then, we used the validation cohort for validation. And group high-risk and low-risk according to the risk score obtained in training cohort. Then, KM survival time analysis, ROC analysis, univariate and multivariate cox regression analysis were performed on the patients in the validation cohort.

### Functional Analysis of the Different Expressed miRNAs

Based on these DENSMs, we utilized three miRNA target gene prediction sites (miRDB, http://mirdb.org; miRTarBase, http://mirtarbase.mbc.nctu.edu.tw; TargetScan, http://www.targetscan.org) to predict the target genes of these 3 miRNAs respectively. Use cytoscape software to display miRNA-target genes network. Gene notology (GO) and the Kyoto Encyclopedia of Genes and Genomes (KEGG) analyses were performed by applying the “clusterProfiler” package (Yu et al., 2012). We further analyzed and validated some miRNA-targeted genes’ expression levels in the GEO database (GSE17025). Further, we analyzed the survival time of some miRNA-targeted genes in the TCGA database.

### Statistical Analysis

In order to compare the difference in miRNA expression levels between normal and EC tissues, we used a one-way analysis of variance. However, when comparing categorical variables, we chose to use the Pearson chi-square test. The Kaplan-Meier method of two-sided log-rank test was used to compare the OS of subgroups of patients. Univariate and multivariate Cox regression models are used to evaluate the independent prognostic value of risk models. All statistical analyses were accomplished with R software (v4.0.5).

## Results

### Identification of Differentially Expressed Necroptosis-Related miRNAs Between Normal and Tumor Tissues

**FIGURE 1:**
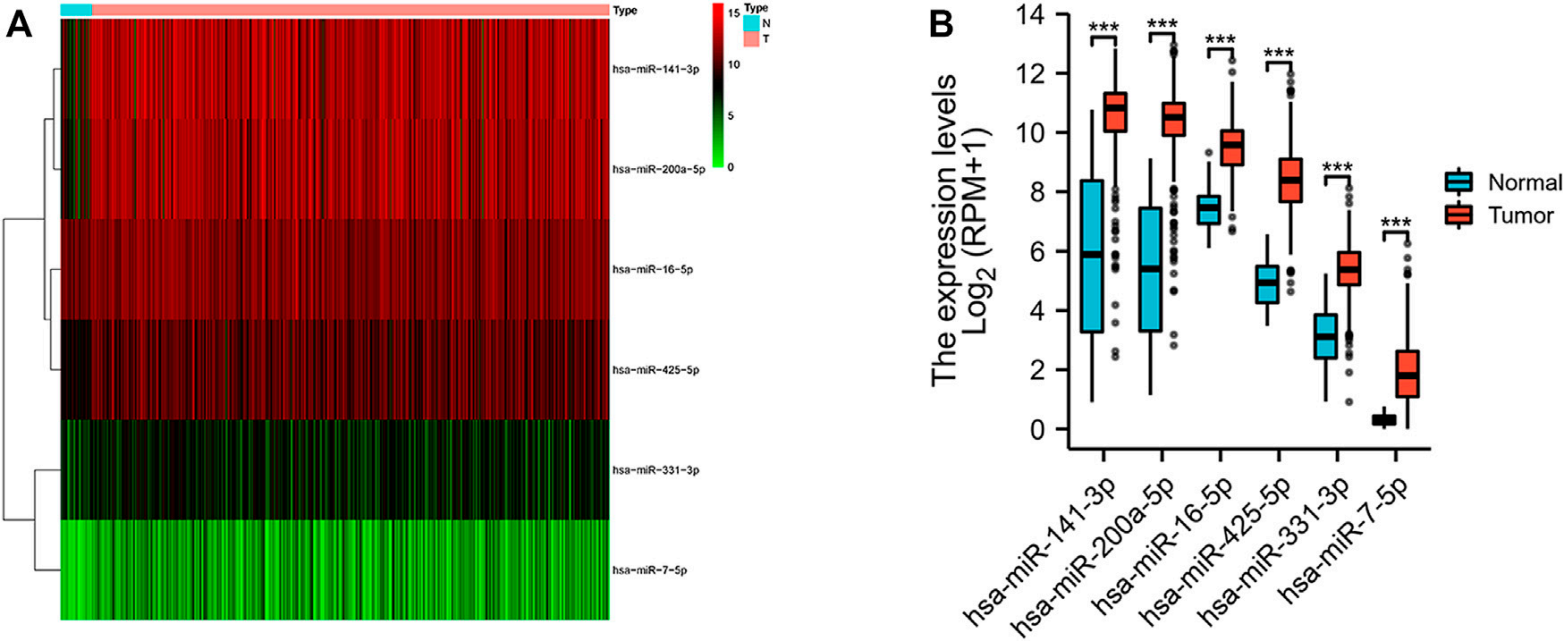
Expressions of the six differentially expressed necroptosis-related miRNAs. **(A)** Heatmap (green: low expression level; red: high expression level) of the differentially expressed necroptosis-related miRNAs between the normal (N, brilliant blue) and the tumor tissues (T, red). **(B)** Box plot of the differentially expressed necroptosis-related miRNAs between the normal (blue) and the tumor tissues (red). ***p < 0.001.

We obtained a total of 33 normal tissues and 546 tumor tissues in the TCGA database, and compared the expression levels of 13 miRNAs related to necroptosis. We found out six differentially expressed necroptosis-related miRNAs (*p* < 0.05). Among them, 6 miRNAs (miR-141-3p, miR-200a-5p, miR-16-5p, miR-425-5p, miR-331-3p, and miR-7-5p) were all enriched in the tumor group. The expression levels of these miRNAs were shown as a heat map (Figure 1A **)** and box plots (Figure 1B).

### Development of the miRNA Prognostic Model

**FIGURE 2:**
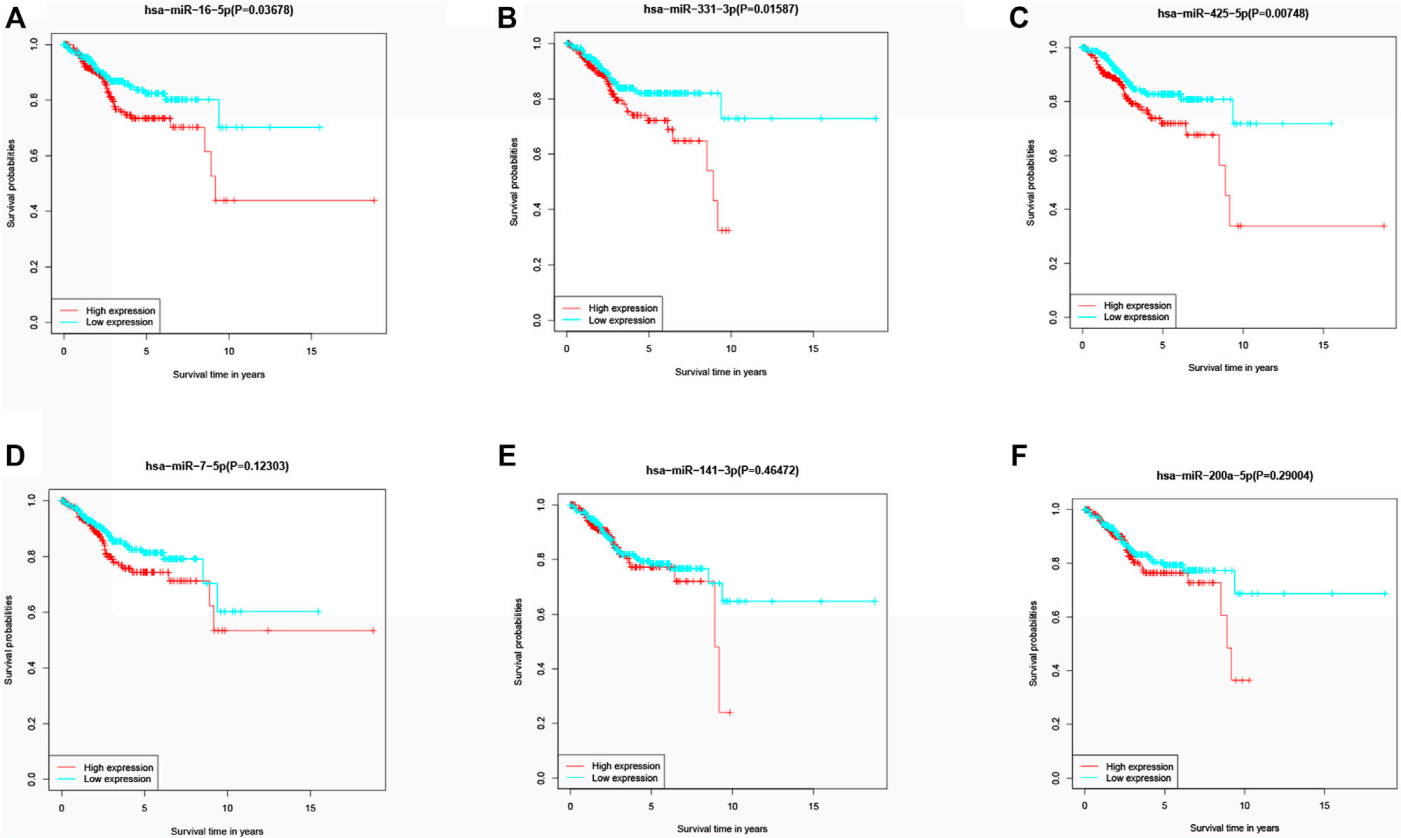
Kaplan–Meier curves of the OS for six miRNAs. **(A)** Kaplan–Meier curves of the OS for miR-16-5p. **(B)** Kaplan–Meier curves of the OS for miR-331-3p. **(C)** Kaplan–Meier curves of the OS for miR-425-5p. **(D)** Kaplan–Meier curves of the OS for miR-7-5p. **(E)** Kaplan–Meier curves of the OS for miR-141-3p. **(F)** Kaplan–Meier curves of the OS for miR-200a-5p.

**FIGURE 3:**
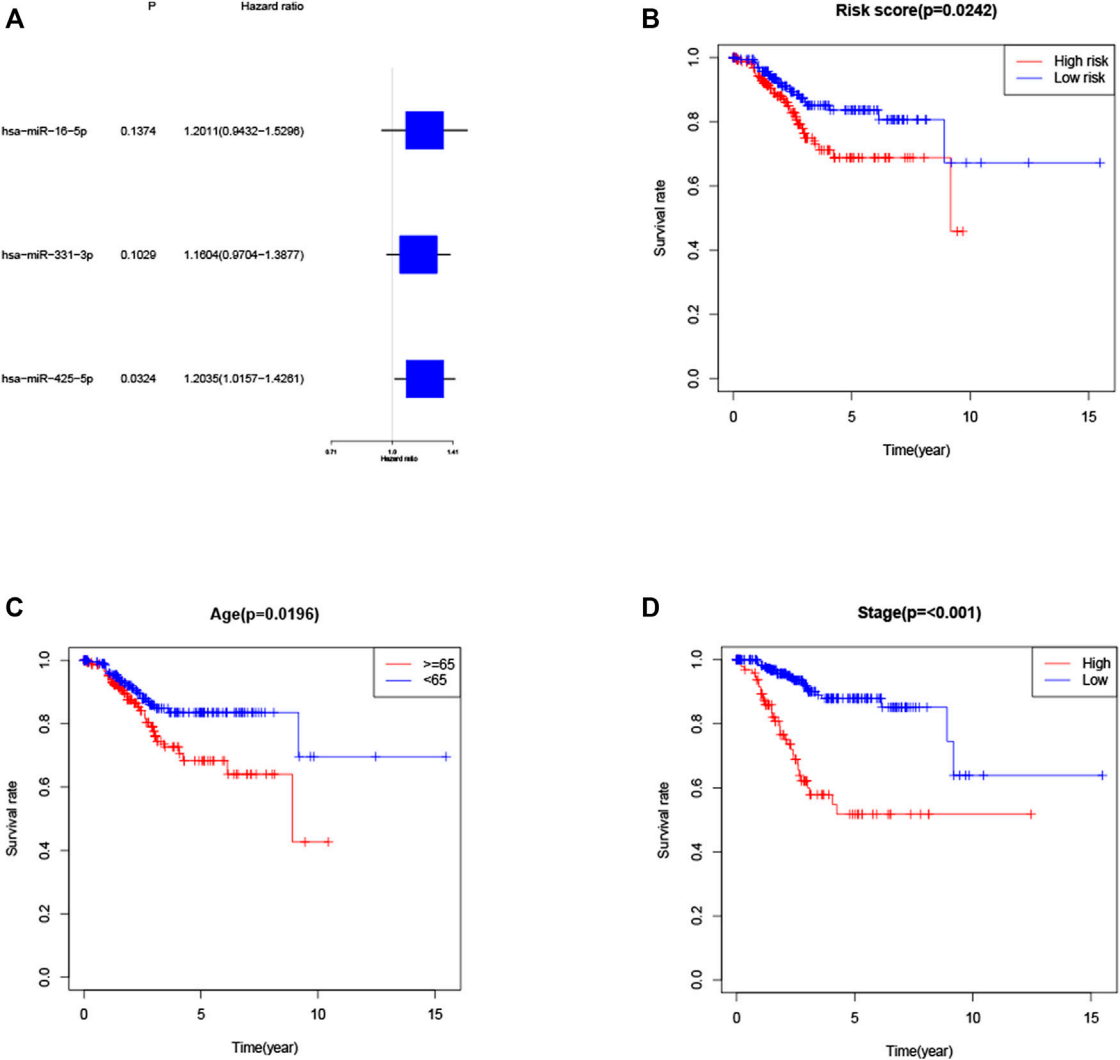
Construction of the risk score model. **(A)** Univariate cox regression analysis of OS for three DENSMs in TCGA cohort. **(B)** Kaplan–Meier curves for the OS of patients in the high- and low-risk groups in training cohort. **(C)** Kaplan–Meier curves for the OS of patients in the high- and low-age groups in training cohort. **(D)** Kaplan–Meier curves for the OS of patients in the high- and low-stage groups in training cohort.

**FIGURE 4:**
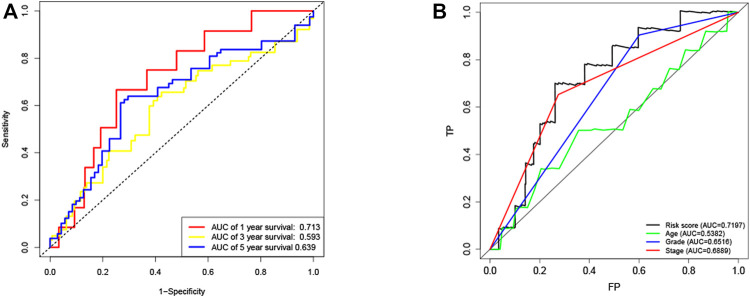
ROC curves of the risk score **(A)** ROC curves demonstrated the predictive efficiency of the risk score at 1, 3 and 5 years in train cohort. **(B)** ROC curves demonstrated the predictive efficiency of the risk score, age, grade and stage at 1 year in training cohort.

KM method was applied to assess survival time data of six differentially expressed miRNAs. Analysis demonstrated higher expression levels of miR-16-5p (*p* = 0.03678, Figure 2A), miR-331-3p (*p* = 0.01587, Figure 2B) and miR-425-5p (*p* = 0.00748, Figure 2C) were all correlated with shorter survival time. In addition, there was no significant correlation between the expression levels of miR-7-5p (Figure 2D), miR-141-3p (Figure 2E) and miR-200a-5p (Figure 2F) and survival time. Preliminary analysis of three DENSMs using univariate Cox regression analysis. The miR-425-5p met the criteria of *p* < 0.05 which increased the risk with HR = 1.2035 (1.0157–1.4261) (*p* = 0.0324, Figure 3A). In addition to this, the miR-16-5p and miR-331-3p met the criteria of *p* < 0.15 which increase the risk with HR = 1.2011 (0.9432–1.5296) and 1.1604 (0.9704–1.3877) respectively (Figure 3A). Then we divided the patient data of TCGA into two groups according to the ratio of 2:1, one is training cohort for building the prognostic model, and the other is validation cohort for model validation. Based on the above 3 DENSMs, we constructed a prognostic model. The risk score was calculated as follows: risk score = (−0.04199*miR-16-5p exp.) + (0.10270*miR-331-3p exp.) + (0.06461 *miR-425-5p exp.). According to the median score calculated using the risk score formula, 364 patients in training cohort were divided equally into a low-risk and a high-risk subgroup. There is a significant difference between the OS time of patients in the low-risk group and the high-risk group (*p* = 0.0242, Figure 3B) in training cohort. In addition, age (*p* = 0.0196, Figure 3C) and stage (*p* < 0.001, Figure 3D) also showed a correlation with survival time in training cohort. Using time-dependent ROC analysis to assess the sensitivity and specificity of the predictive model, we found that the AUC were 0.713 for 1 year, 0.593 for 3 years and 0.639 for 5 years (Figure 4A) in training cohort. Then we used ROC analysis to evaluate the sensitivity and specificity for risk score, age, grade and stage, we found that the AUC were 0.7197 for risk score, 0.5382 for age, 0.6516 for grade and 0.6889 for stage at 1 year (Figure 4B) in training cohort.

### Independent Prognostic Value of the Risk Model

**FIGURE 5:**
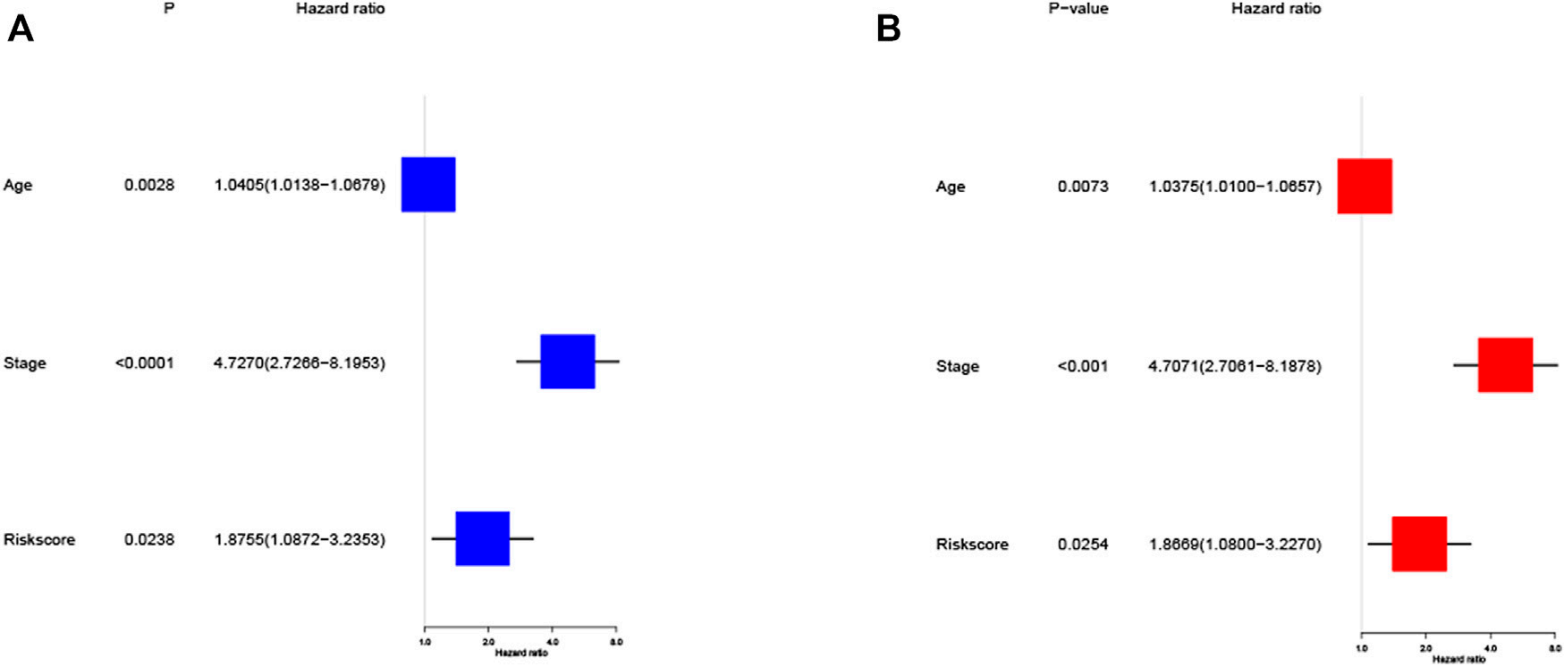
Cox regression analysis of the risk score in training cohort. **(A)** Univariate regression analysis of the risk score in training cohort. **(B)** Multivariate regression analysis of the risk score in training cohort. (Stage: FIGO stage, I and II vs. III and IV).

Univariate and multivariate Cox regression analysis are used to assess whether the risk score derived from the model can be used as an independent predictor. Univariate Cox regression analysis showed that risk score was a predictor of low survival rate in training cohort (HR = 1.8755, 95% CI: 1.0872–3.2353, *p* = 0.0238, Figure 5A). Multivariate regression analysis also implied that risk score was an independent prognostic factor after adjusting for other confounding factors (HR = 1.8669, 95% CI: 1.0800–3.2270, *p* = 0.0254, Figure 5B) for patients with EC in training cohort.

### Validation of the Risk Signature

**FIGURE 6:**
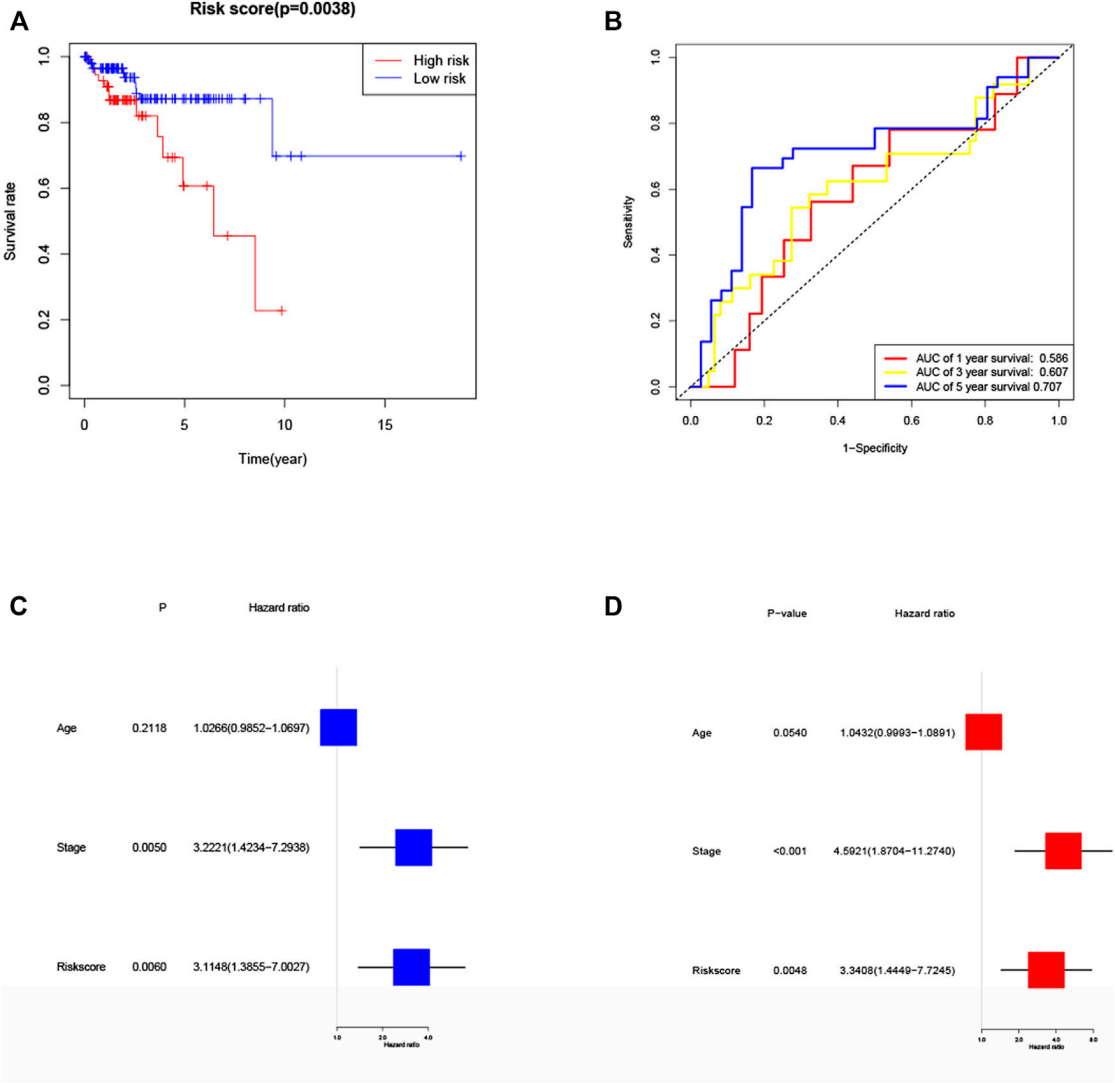
Validation of the risk model in validation cohort. **(A)** Kaplan–Meier curves for the OS of patients in the high- and low-risk groups in validation cohort. **(B)** ROC curves demonstrated the predictive efficiency of the risk score at 1, 3 and 5 years in validation cohort. **(C)** Univariate regression analysis of the risk score in validation cohort. **(D)** Multivariate regression analysis of the risk score in validation cohort. (Stage: FIGO stage, I and II vs. III and IV).

To further validate the risk model, we used the validation cohort for validation. Based on the risk score of the training cohort, 182 patients in validation cohort were classified into the high- and low-risk group. KM analysis showed a significant difference between the OS time of patients in the low-risk group and the high-risk group (*p* = 0.0038, Figure 6A) in validation cohort. ROC analysis showed that the AUC were 0.586 for 1 year, 0.607 for 3 years and 0.707 for 5 years (Figure 6B) in validation cohort. Univariate Cox regression analysis showed that risk score was a predictor of low survival rate in validation cohort (HR = 3.1148, 95% CI: 1.3855–7.0027, *p* = 0.0060, Figure 6C). Multivariate regression analysis also implied that risk score was an independent prognostic factor after adjusting for other confounding factors (HR = 3.3408, 95% CI: 1.4449–7.7245, *p* = 0.0048, Figure 6D) in validation cohort.

### Functional Analyses

**FIGURE 7:**
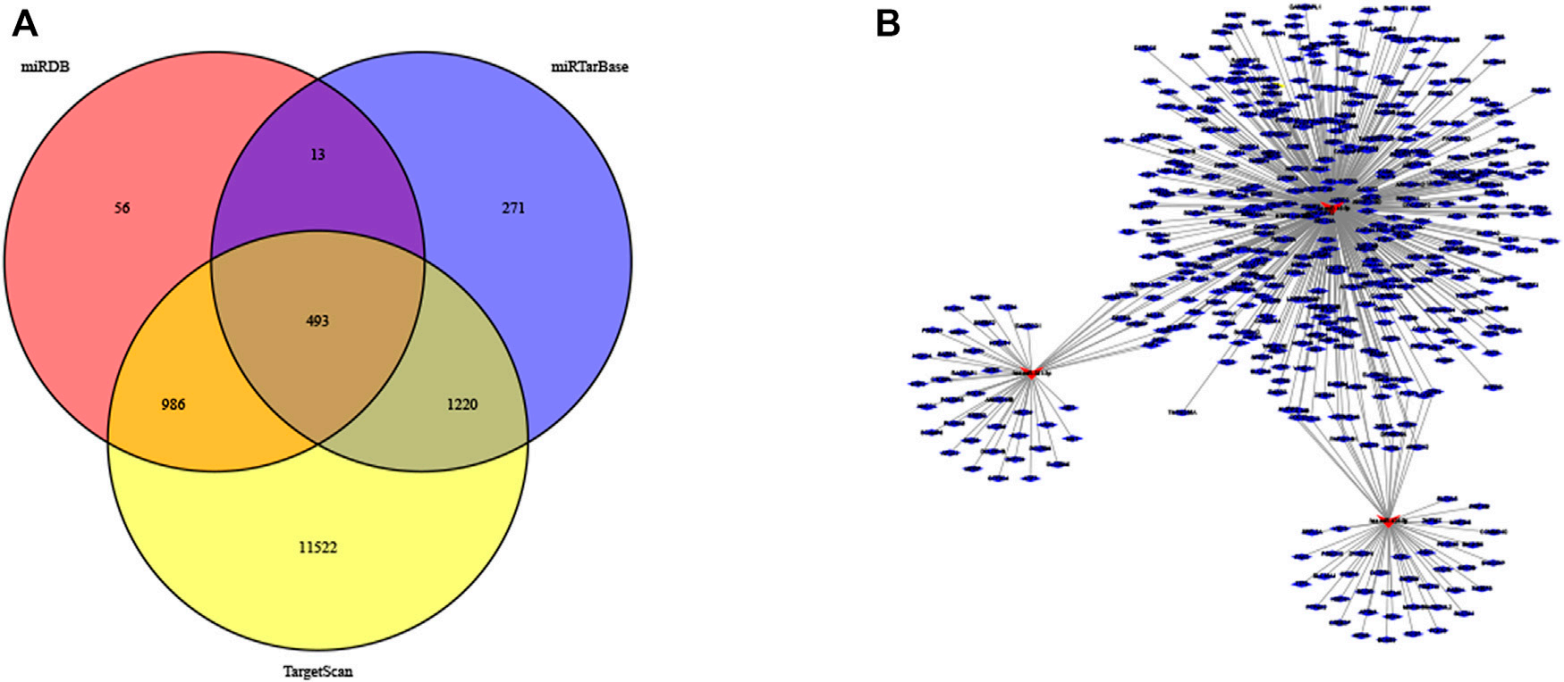
MiRNA target genes of the three DENSMs. **(A)** The Venn diagram of DENSMs by three prediction websites (miRDB, miRTarBase, TargetScan). **(B)** The network of DENSMs made by Cytoscape.

**FIGURE 8:**
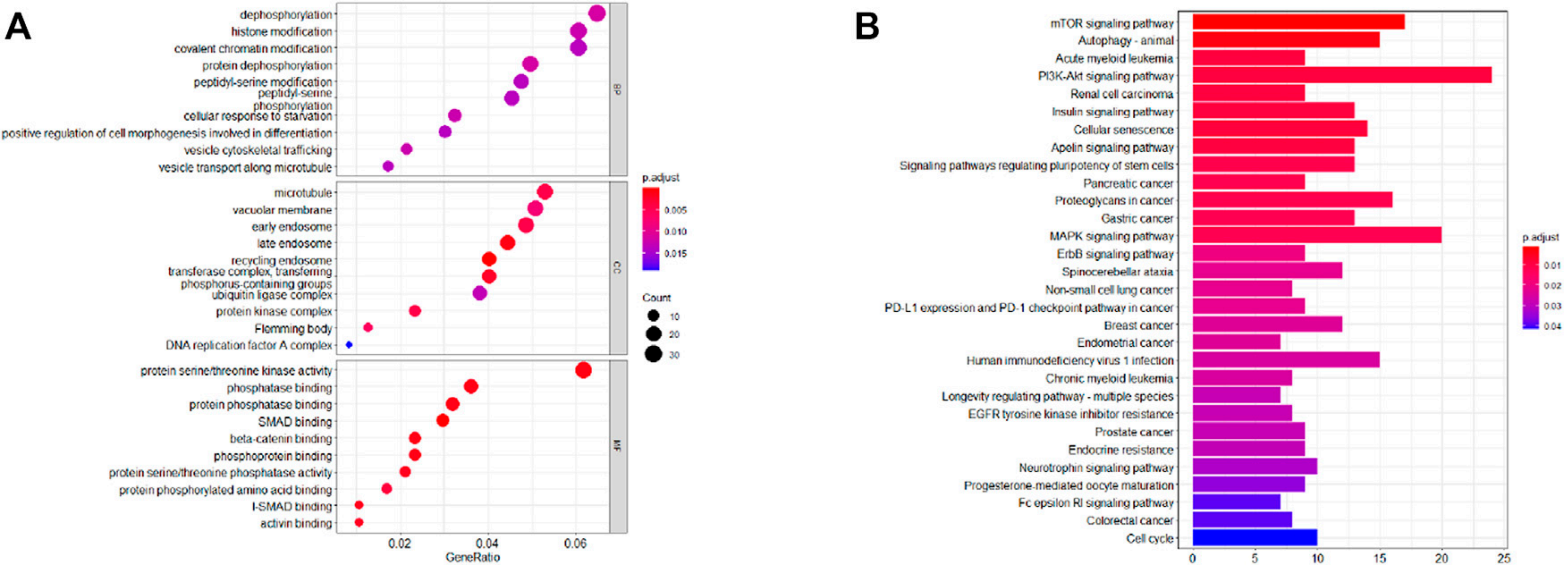
Functional analysis based on the MiRNA target genes of the three DENSMs. **(A)** Bubble graph for GO enrichment for DENSMs target genes (the bigger bubble means the more genes enriched, and the increasing depth of red means the differences were more obvious; q-value: the adjusted *p*-value). **(B)** Barplot graph for KEGG pathways for DENSMs target genes (the longer bar means the more genes enriched, and the increasing depth of red means the differences were more obvious).

**FIGURE 9:**
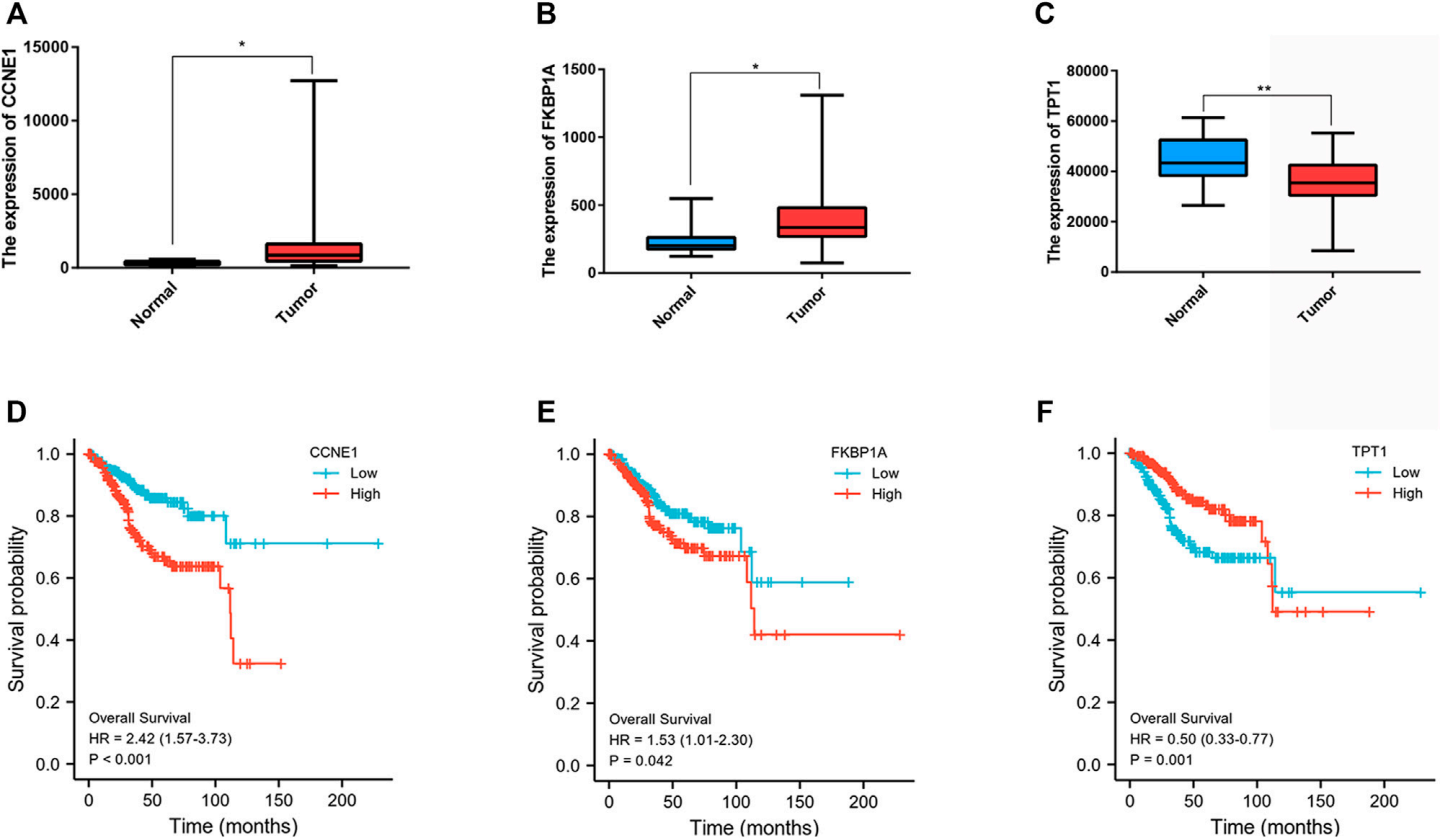
Expression and survival analysis of three MiRNA target genes. **(A)** Box plot of the differentially expressed MiRNA target genes (CCNE1) between the normal (blue) and the tumor tissues (red) in GEO database. **(B)** Box plot of the differentially expressed MiRNA target genes (FKBP1A) between the normal (blue) and the tumor tissues (red) in GEO database. **(C)** Box plot of the differentially expressed MiRNA target genes (TPT1) between the normal (blue) and the tumor tissues (red) in GEO database. **(D)** Kaplan–Meier curves of the OS for CCNE1 in GEO database. **(E)** Kaplan–Meier curves of the OS for FKBP1A in GEO database. **(F)** Kaplan–Meier curves of the OS for TPT1in GEO database. *p < 0.05; **p < 0.01.

Further examine the differences of functions and pathways in the three DENSMs, we utilized three miRNA target gene prediction sites (miRDB, http://mirdb.org; miRTarBase, http://mirtarbase.mbc.nctu.edu.tw; TargetScan, http://www.targetscan.org) to predict the target genes of these three miRNAs respectively. Finally, through these three DENSMs, we found 493 target genes (Figure 7A). The network constructed by the three DENSMs and their 493 target genes were exhibited (Figure 7B). Then based on these 493 target genes, GO enrichment analysis and KEGG pathway analysis were performed. GO analysis is divided into three parts, namely biological process, cell composition and molecular function analysis. Biological processes are mainly related to dephosphorylation, histone modification and covalent chromatin modification. Cellular components are mainly related to microtubules, vacuolar membranes and early endosomes. The molecular function is mainly related to protein serine/threonine kinase activity (Figure 8A). The KEGG results indicated that the target genes were mainly related with the PI3K-Akt signaling pathway and MAPK signaling pathway (Figure 8B). This means that the DENSMs are likely to be related to these functional pathways. We further analyzed and validated some miRNA-targeted genes. We obtained 91 EC patients and 12 normal controls from the GEO database (GSE17025) for expression analysis. We show the expression of three differentially expressed miRNA-targeted genes, CCNE1 (Figure 9A), FKBP1A (Figure 9B) and TPT1 (Figure 9C). Further, we analyzed the survival time of these three genes in the TCGA database. KM analysis showed that CCNE1 (Figure 9D), FKBP1A (Figure 9E) and TPT1 (Figure 9F) were significantly correlated with OS.

## Discussion

Although there have been some articles using genetic models to predict the prognosis of EC patients (Wang H. et al., 2020; Zhang et al., 2020a; Wang X. et al., 2021; Weijiao et al., 2021), there is no literature that systematically uses necroptosis-related miRNAs to predict the prognosis of EC patients. As far as we know, this study is the first to systematically use necroptosis-related miRNAs to predict the prognosis of EC patients. In this study, we adopted three DENSMs (miR-16-5p, miR-331-3p and miR-425-5p) to construct prognostic models. As to our prognostic models, KM survival analysis, univariate Cox regression, multivariate Cox regression and the AUC all illustrated our models had a great predictive performance. Additionally, the risk scores of the OS model could be considered as independent predictive indicators. At the same time, we also analyzed the performance of clinical variables including age, grade, and stage in KM analysis and ROC curve analysis. It can be seen that stage is still a good prognostic indicator. In addition, we performed further validation of the prognostic model using the validation cohort. In the validation cohort, the predictive effect of the prognostic model in EC was very good, both in KM prognostic analysis, univariate and multivariate cox regression and ROC curve analysis. This model can provide a new reference for prognostic risk stratification assessment and treatment strategy selection for EC patients.

Necroptosis is a new type of programmed necrosis (Galluzzi et al., 2017; Choi et al., 2019). It has two opposite effects in antitumor and protumor (Su et al., 2015; Tang et al., 2020). In the present study, we successfully constructed a prognostic model based on three DENSMs. These three DENSMs have been found to be related to ECs. MiR-331-3p has been reported to play an important role in the EMT of ECs of which has been shown to related to survival in our study (Zmarzły et al., 2021). Nikola Zmarzły et al. found that the Wnt signaling is disrupted in endometrial cancer, which may be due to miR-331-3p activity (Zmarzły et al., 2021). MiR-16-5p may also play an important role in the occurrence and development of EC. Piotr Januszyk et al. found that significant changes in the expression of miR-16 with Drug Resistance in Endometrial Cancer Cells Treated with Salinomycin (Januszyk et al., 2021). Xiyin Wang et al. found that the expression of miR-16 has also changed significantly with Pten and Dicer1 lost in the mouse uterus causes poorly differentiated endometrial adenocarcinoma (Wang X. et al., 2020). Priti Kumari et al. reported when looking for Diagnostic potential of differentially regulated microRNAs among endometriosis, endometrioid ovarian cancer, and endometrial cancer, they found that miR-16 can be used as one of the important markers (Kumari et al., 2021). In addition, GO functional enrichment and KEGG signaling pathway analysis showed that differentially expressed necroptosis-related miRNAs were involved in some cancer-related signaling pathway such as dephosphorylation, histone modification, covalent chromatin modification, the PI3K-Akt signaling pathway and MAPK signaling pathway. In summary, these differently expressed necroptosis-related miRNAs are involved in many important biological functions and signaling pathways in connection with ECs. At the same time, we analyzed the expression levels of some miRNA target genes in the GEO database, and found that some of them had obvious expression differences. We then further analyzed the prognosis of these differentially expressed miRNA target genes in the TCGA database, and found that some of them also had significant prognostic differences. We showed three representative miRNA target genes in the results, CCNE1, FKBP1A, TPT1. Xu et al. found that CCNE1 copy number is a biomarker for response to combination WEE1-ATR inhibition in endometrial cancer (Xu et al., 2021). FKBP1A has been shown to have a clear role in other cancers including prostate cancer and head and neck squamous cell carcinoma (Zhang et al., 2019b; Patel et al., 2022). Zhang et al. found that TPT1 was a hub gene in EC and there were some studies had reported that TPT1 had role in some cancers (Zhang and Wang, 2019a; Zhang and Wang, 2020b; Wang N. et al., 2021). These miRNA target genes can be a direction guide for our future EC research to better explore the relationship between EC and necroptosis and its pathogenesis.

There are some limitations in this study. First of all, all the data in this study came from TCGA database, and the total sample size was relatively insufficient. Larger clinical samples are needed to validate the conclusions of this study. Secondly, due to the lack of more data on clinical variables, we are unable to conduct a more comprehensive clinical analysis. Thirdly, the molecular mechanism of necroptosis affecting the prognosis of endometrial cancer patients and its significance for clinical translational therapy need to be further studied.

## Conclusion

In this study, a risk prediction model of necroptosis-related miRNAs was successfully constructed, which can effectively predict the prognosis of EC patients. This model can provide biomarkers and therapeutic targets for accurate treatment of endometrial cancer in the future.

## Data Availability

The original contributions presented in the study are included in the article/Supplementary Material, further inquiries can be directed to the corresponding author.
